# Transcriptional profile of *Taxus chinensis* cells in response to methyl jasmonate

**DOI:** 10.1186/1471-2164-13-295

**Published:** 2012-07-02

**Authors:** Shu-tao Li, Peng Zhang, Meng Zhang, Chun-hua Fu, Chun-fang Zhao, Yan-shan Dong, An-yuan Guo, Long-jiang Yu

**Affiliations:** 1 Institute of Resource Biology and Biotechnology, Department of Biotechnology, College of Life Science and Technology, Huazhong University of Science and Technology, Wuhan, China; 2 Key Laboratory of Molecular Biophysics Ministry of Education, Huazhong University of Science and Technology, Wuhan, China; 3 Department of Biomedical Engineering, College of Life Science and Technology, Huazhong University of Science and Technology, Wuhan, China

## Abstract

**Background:**

Methyl jasmonate (MeJA) has been successfully used as an effective elicitor to enhance production of taxol and other taxanes in cultured *Taxus* cells. However the mechanism of MeJA-mediated taxane biosynthesis remains unclear. Genomic information for species in the genus *Taxus* is currently unavailable. Therefore, information about the transcriptome of *Taxus* cells and specifically, description of changes in gene expression in response to MeJA, is needed for the better exploration of the biological mechanisms of MeJA-mediated taxane biosynthesis.

**Results:**

In this research, the transcriptome profiles of *T. chinensis* cells at 16 hours (T16) after MeJA treatment and of mock-treated cells (T0) were analyzed by “RNA-seq” to investigate the transcriptional alterations of *Taxus* cell in response to MeJA elicitation. More than 58 million reads (200 bp in length) of cDNA from both samples were generated, and 46,581 unigenes were found. There were 13,469 genes found to be expressed differentially between the two timepoints, including all of the known jasmonate (JA) biosynthesis/JA signaling pathway genes and taxol-related genes. The qRT-PCR results showed that the expression profiles of 12 randomly selected DEGs and 10 taxol biosynthesis genes were found to be consistent with the RNA-Seq data. MeJA appeared to stimulate a large number of genes involved in several relevant functional categories, such as plant hormone biosynthesis and phenylpropanoid biosynthesis. Additionally, many genes encoding transcription factors were shown to respond to MeJA elicitation.

**Conclusions:**

The results of a transcriptome analysis suggest that exogenous application of MeJA could induce JA biosynthesis/JA signaling pathway/defence responses, activate a series of transcription factors, as well as increase expression of genes in the terpenoid biosynthesis pathway responsible for taxol synthesis. This comprehensive description of gene expression information could greatly facilitate our understanding of the molecular mechanisms of MeJA-mediated taxane biosynthesis in *Taxus* cells.

## Background

Taxol (generic name paclitaxel, Bristol-Myers Squibb), isolated from the bark of *Taxus brevifolia*[1], is a widely employed anticancer drug. Production of taxol directly from yew trees remains a challenging problem due to the limited resources of *Taxus sp.*

Cultured *Taxus* cells as a renewable and sustainable system are a promising production route for taxol and related taxanes [2-4]. However, the low abundance of taxol in cell cultures has limited their industrial application [3,4]. Methyl jasmonate (MeJA), as an inducer of jasmonates (JAs), regulates a diverse set of physiological and developmental processes [5], and addition of MeJA can significantly induce the production of taxol and related taxanes in *Taxus sp.* suspension cultures [2-4]. Several secondary metabolites were also found to accumulate in plant cell cultures upon MeJA elicitation, such as terpenoid indole alkaloids in *Catharanthus roseus* cells [6] and nicotine/phenylpropanoid conjugate in *Nicotiana tabacum* cells [7-9]. In *Catharanthus roseus*, the MeJA-responsive expression of terpenoid indole alkaloids biosynthesis genes has been shown to be controlled by a transcription factor cascade consisting of the bHLH protein CrMYC2’s regulation of ORCA gene expression, and thus the AP2/ERF-domain transcription factors ORCA2 and ORCA3, which in turn regulate a series of terpenoid indole alkaloids biosynthesis genes [6,10]. In Tobacco, the AP2/ERF and bHLH transcription factors cooperatively mediate jasmonate-elicited nicotine biosynthesis, which via the JA induced signaling cascade leads to increased nicotine biosynthesis [11,12]. However, though some mechanisms of the JA-elicited biosynthesis of secondary metabolites have been elucidated, the detailed biological mechanism of MeJA stimulation of taxane production and concomitant transcriptome changes associated with response to MeJA remain poorly understood.

RNA-seq is a high-throughput and cost-effective DNA sequencing technology that is not dependent on a prior description of the genomic sequence of the target species [13-15]. In addition, RNA-seq is capable of detecting low abundant transcripts [14,16], and as it produces millions of short cDNA reads, the technology also provides information about the transcriptional structure and the gene-expression profiles [15,16]. Recent research has demonstrated that RNA-Seq is not only well-suited for surveying the complexity of transcription, but also for discovering genes and comparing gene expression profiles in eukaryotes [13-18].

In this research, *T. chinensis* cells treated with MeJA for 16 h (T16) and the control cells mock-treated (T0) were analyzed by RNA-seq to describe the transcriptome and reveal transcriptional profiles in response to MeJA induction in *T. chinensis* cells. Despite there being no complete genomic sequence of *T. chinensis,* 58 million reads (200 bp in length) of high-quality DNA sequence were generated using Illumina technology, a total of 46,581 unigenes in numerous functional categories were annotated in a eukaryote without the prior genome information, and 13,469 genes were found to be differentially expressed between the two treatments. These assembled and annotated transcriptome sequences and gene expression profiles were analyzed to provide insight into the transcriptional changes in response to MeJA in *T. chinensis* cells, which should help to elucidate the molecular mechanisms of MeJA-mediated taxane biosynthesis and MeJA-modulated network formation.

## Results

### Illumina sequencing and sequence assembly

Total RNAs were respectively extracted from the MeJA-treated *T. chinensis* cells for 16 h (T16) and the mock-treated cells with an equal volume of ethanol (T0), and the poly (A) + RNA from the two samples was isolated, sheered into smaller fragments, and reverse-transcribed to cDNA. A small portion of each library was cloned to determine the quality of the cDNAs, and then the cDNA libraries were subjected to high throughput parallel sequencing with Solexa/Illumina technology to investigate the transcriptome information and characterize changes in gene expression responding to MeJA induction.

In total, 29,459,951 reads of 200 bp sequence were generated from the T0 sample (Table 1); the Q20 percentage (percentage of bases whose quality was larger than 20 in clean reads), N percentage, and GC percentage are 93.85%, 0.01% and 45.69%, respectively. 29,896,420 reads were generated from the T16 sample (Table 1); the Q20 percentage, N percentage, and GC percentage are 93.74%, 0.02% and 44.96% for T16, respectively. These reads were randomly assembled to produce 109,489 contigs with an N50 of 423 bp (i.e. 50% of the assembled bases were incorporated into contigs 423 bp or longer) for T0 and 108,772 contigs with an N50 of 407 bp for T16 (Table 1, Additional file 1). Although most contigs were between 100 and 200 bp, 13.07% reads of T0 (14,309 contigs) and 12.45% reads of T16 (13,544 contigs) were greater than 500 bp in length (Additional file 1).

**Table 1:** The statistics of RNA-seq data

	**Reads**	**Contigs**	**Scaffolds**	**Unigenes**
T0	29,459,951	109,489	61,703	39,176
T16	29,896,420	108,772	60,601	38,713

The contigs further assembled with paired-end joining and gap-filling to produce 61,703 scaffolds with an N50 of 839 bp (7,607 of which larger than 1,000 bp) for T0 and 60,601 scaffolds with an N50 of 812 bp (7,101 of which larger than 1,000 bp) for T16 (Table 1, Additional file 2). These scaffolds were respectively clustered with TGICL software [19] to generate 39,176 unigenes for T0 and 38,713 for T16, totalling 46,581 assembled unigenes (Table 1). These results indicate that the assembly and contig joining succeeded in processing a large amount of short reads from *T. chinensis* cell samples with relatively little redundancy.

**Figure 1:**
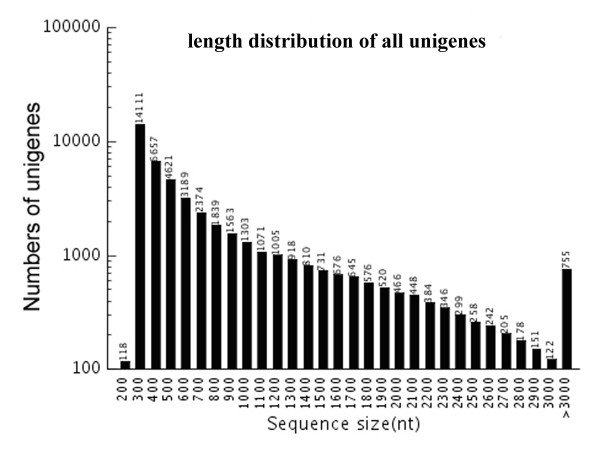
Assembled unigenes length distribution. Distribution of all assembled unigenes lengths.

Among the 46,581 assembled unigenes, 21,111 unigenes were ≥500 bp and 10,823 were ≥1,000 bp, with a mean unigenes length of 744 bp and an N50 of 1198 bp. The size distribution for these unigenes was shown in Figure 1. We analyzed the ratio of the gap’s length to the length of assembled unigenes. The results revealed that the majority of the unigenes, which accounted for 85.47% of total unigenes, showed gap lengths that were less than 5% of the total length, suggesting that our sequence data was highly suitable for further analysis. The transcriptome data of *T. chinensis* cells were submitted in the datasets of Gene Expression Omnibus (GEO) (accession number GSE28539).

### Functional annotation of predicted proteins

**Figure 2:**
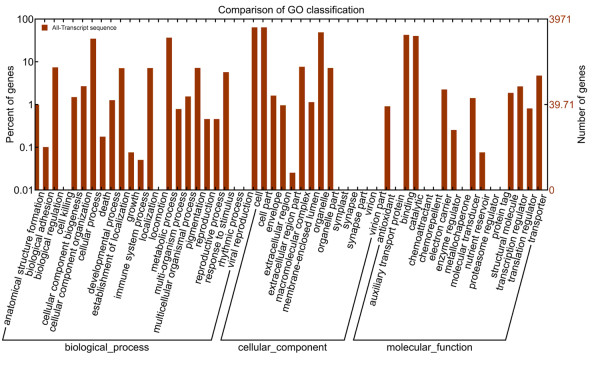
**Go annotation of all unigenes.** Annotated sequences were classified into ‘Biological Process’, ‘Molecular Function’ and ‘Cellular Component’ groups and 54 subgroups.

**Figure 3:**
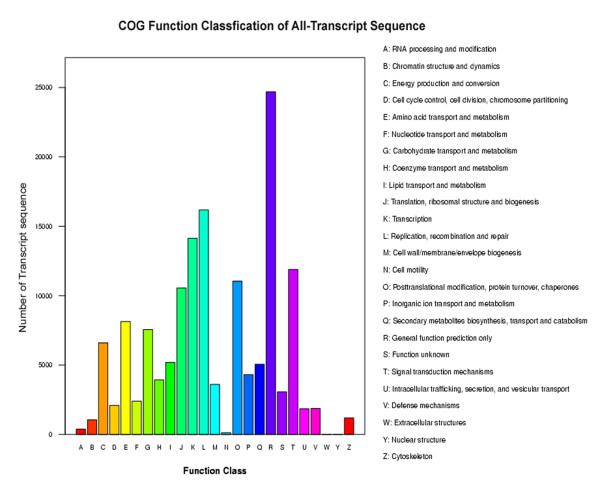
**COG function classification of all-unigenes.** Out of 25,812 NR hits, 5,812 sequences have a COG classification among the 25 categories. A, RNA processing and modification; B, Chromatin structure and dynamics; C, Energy production and conversion; D, Cell cycle control, cell division, chromosome partitioning; E, Amino acid transport and metabolism; F, Nucleotide transport and metabolism; G, Carbohydrate transport and metabolism; H, Coenzyme transport and metabolism; I, Lipid transport and metabolism; J, Translation, ribosomal structure and biogenesis; K, Transcription; L, Replication, recombination and repair; M, Cell wall/membrane/envelope biogenesis; N, Cell motility; O, Posttranslational modification, protein turnover, chaperones; P, Inorganic ion transport and metabolism; Q, Secondary metabolites biosynthesis, transport and catabolism; R, General function prediction only; S, Function unknown; T, Signal transduction mechanisms; U, Intracellular trafficking, secretion, and vesicular transport; V, Defense mechanisms; W, Extracellular structures; Y, Nuclear structure; Z, Cytoskeleton.

Functional annotation of the *T. chinensis* transcriptome sequences was first performed against the non-redundant protein database (NR) at NCBI with a cut-off E-value of 10^-5^. Due to the lack of *T. chinensis* genome information and the relatively short length of distinct gene sequences, only 25,812 unigenes (55.41% of all distinct sequences) were shown to be annotated with NR (Additional file 3). 34.46% of the unigenes shorter than 500 bp (8778/25470) could achieve significant BLAST scores in the NR database. In contrast, the proportion of unigenes with significant BLAST scores increased to: 65.78% for query sequences between 500 and 1,000 bp (6767/10288); 90.7% for query sequences between 1,000 and 1,500 bp (4113/4546); 96.71% for query sequences between 1,500 and 2,000 bp (2785/2880); and 99.18% for query sequences ≥2,000 bp (3369/3397). These results indicated that the proportion of sequences with functional annotation in the NR database is greater among the longer assembled sequences. The Gene Ontology (GO) terms were assigned to classify the function of the predicted *T. chinensis* unigenes. Based on the sequence homology, the 25,812 annotated unigenes were then analyzed with Blast2GO for GO classification [20] to generate 3,971 unigenes, which were categorized into 54 functional groups by WEGO [21] in the three categories of biological processes, cellular components and molecular function (Figure 2). Seven GO terms: “cell”, “cell part”, “organelle”, “binding”, “metabolic process”, “catalytic” and “cellular process” were predominantly represented (Figure 2). To further evaluate the completeness of our transcriptome library and the effectiveness of our annotation process, we searched the annotated sequences for the genes involved in Clusters of Orthologous Groups (COGs) classifications. All of the 46,581 unigenes were aligned to the COGs database to predict and classify possible functions. 5,812 unique sequences had a COGs classification, comprising in total 25 categories. In these 25 categories, R category (general function prediction only) is the largest group, followed by L category (replication, recombination and repair) and K category (transcription) (Figure 3).

Moreover, to identify the biological pathways that were actived in *T. chinensis* cells, all unigenes were mapped in the Kyoto Encyclopedia of Genes and Genomes (KEGG) database and 12,640 unigenes were assigned to 124 KEGG pathways. Among them, the three most representative pathways were metabolic pathways (2,857 members), spliceosome (805 members) and biosynthesis of plant hormones (644 members) (Additional file 4). The annotations of these unigenes showed a significant transcriptional complexity and provided valuable gene expression information in the transcriptome of *T. chinensis* cells.

### Differentially expressed gene analysis and qRT-PCR validation

**Figure 4:**
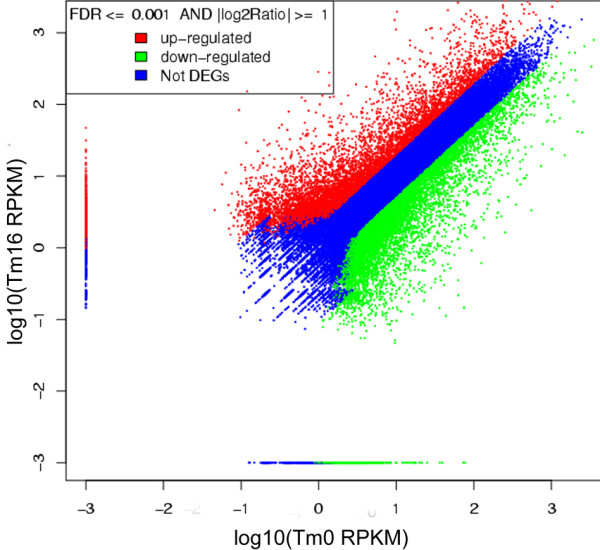
**Expression level T0 vs T16.** The expression level of T0 vs Tm16. The red points represent up-regulated unigenes, the green points represents down-regulated unigenes, and the blue points represent the non-DEGs.

Normalized expression value of genes were calculated by a RPKM (Reads Per kb per Million reads) method [22], and differentially expressed genes (DEGs) between two samples were identified by FDR (False Discovery Rate) method according to Audic et al. [23]. A total of 13,469 genes were shown to be differentially expressed in response to MeJA elicitation (Figure 4). Compared with the control (T0), the expression levels of 6,347 DEGs were up-regulated and those of 7,122 DEGs were down-regulated in MeJA-treated *T. chinensis* cells (T16) (Additional file 5, Additional file 6). Examining the ten most up-regulated and ten of the most down-regulated genes, eight of the up-regulated genes have defined functions, including lipoxygenase, phenylpropanoyl transferase and auxin-responsive family protein, and five down-regulated genes have defined functions, such as RING-H2 finger protein ATL1R and cytochrome P450 monooxygenase.

GO classification analysis of 13,469 DEGs showed that a large number of DEGs were dominant in 7 terms, e.g. “cell part” and “binding” (Additional file 7), and we showed GO categories for up- and down-regulated genes separately (Additional file 8). All DEGs were then mapped in the KEGG database to search for genes involved in metabolic or signal transduction pathways. The two cell lines (T0, T16) were analyzed by KEGG, we found that the proteasome, *Vibrio cholerae* infection and cytosolic DNA-sensing pathway had the most significant changes (Additional file 9). 3,391 DEGs were annotated by KEGG, and this annotation revealed significant enrichment for genes found in metabolic pathways (806 DEGs, 23.77%), biosynthesis of plant hormones (226 DEGs, 6.66%), and biosynthesis of phenylpropanoids (204 DEGs, 6.02%) (Additional file 10). This annotation of genes differentially induced by MeJA will provide a valuable resource for investigating specific processes, functions and pathways responding to MeJA in *T. chinensis* cells. To validate the RNA-Seq data for differential gene expression between the two samples, qRT-PCR was applied to test the expression of 12 randomly selected DEGs. qRT-PCR expression profiles of the 12 genes were found to be consistent with the RNA-Seq data (Additional file 11). These results further supported that the RNA-seq data is reliable.

GO classification analysis of 13,469 DEGs showed that a large number of DEGs were dominant in 7 terms, e.g. “cell part” and “binding” (Additional file 7), and we showed GO categories for up- and down-regulated genes separately (Additional file 8). All DEGs were then mapped in the KEGG database to search for genes involved in metabolic or signal transduction pathways. The two cell lines (T0, T16) were analyzed by KEGG, we found that the proteasome, *Vibrio cholerae* infection and cytosolic DNA-sensing pathway had the most significant changes (Additional file 9). 3,391 DEGs were annotated by KEGG, and this annotation revealed significant enrichment for genes found in metabolic pathways (806 DEGs, 23.77%), biosynthesis of plant hormones (226 DEGs, 6.66%), and biosynthesis of phenylpropanoids (204 DEGs, 6.02%) (Additional file 10). This annotation of genes differentially induced by MeJA will provide a valuable resource for investigating specific processes, functions and pathways responding to MeJA in *T. chinensis* cells. To validate the RNA-Seq data for differential gene expression between the two samples, qRT-PCR was applied to test the expression of 12 randomly selected DEGs. qRT-PCR expression profiles of the 12 genes were found to be consistent with the RNA-Seq data (Additional file 11). These results further supported that the RNA-seq data is reliable.

### The MeJA-responsive activity in jasmonate signaling pathway

Jasmonates (JAs) are plant-specific signaling molecules that activate several defence mechanisms, inducing a massive reprogramming of gene expression [5]. Exogenous MeJA is believed to be a primary regulator of the JA biosynthesis and JA signaling pathways in plants, and has been studied extensively in *Solanum lycopersicumNicotiana tabacum*[24] and *Arabidopsis thaliana*[25].

**Figure 5:**
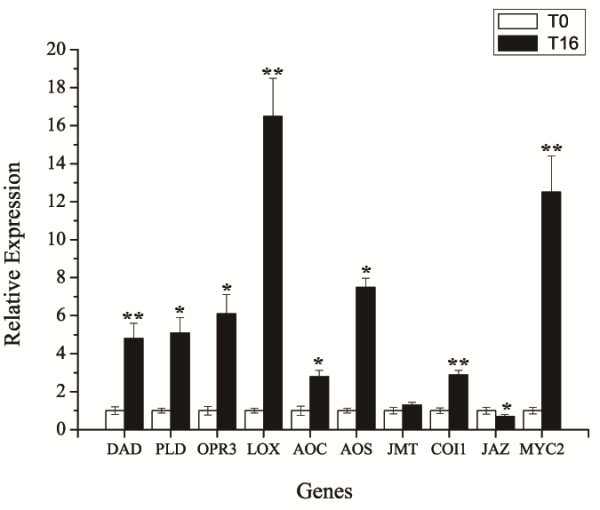
**Relative expression of putative genes involved in JA biosynthesis and JA signaling pathway as determined by qRT-PCR.** qRT-PCR analysis of putative genes involved in JA biosynthesis and JA signaling pathway, *P < 0.05, **P < 0.01. PLD is short for phospholipase D, DAD1 for phospholipase A1, LOXs for lipoxygenases, AOS for allene oxide synthase, AOC for allene oxide cyclase, OPR3 for OPDA reductase, JMT for jasmonic acid carboxyl methyltransferasesignal, COI1 for coronatine-insensitive protein 1, JAZ for jasmonate ZIM-domain protein, and MYC2 is a bHLH transcription factor.

We mapped all the defined genes encoding enzymes for JA biosynthesis and in JA signaling pathways to find highly similar unigenes in the transcriptome of *T. chinensis* cells. Gene expression of seven JA biosynthesis-related genes (PLD, DAD1, LOXs, AOS, AOC, OPR3, JMT) [26] and three JA-signal pathway-related genes (COI1, JAZ, MYC2) [24,25] have been validated by qRT-PCR (Figure 5). The results show that the mRNA levels of these genes were slightly higher than those detected by RNA-Seq. These results suggested that *T. chinensis* cells have a similar MeJA-mediated JA signaling pathway as the JA signaling pathway in *Arabidopsis*. The results clearly confirm that exogenous application of MeJA can regulate JA biosynthesis and JA signaling pathway in *T. chinensis* cells.

### MeJA effects hormone and phenylpropanoids biosynthesis

MeJA can induce jasmonate (JA) biosynthesis and steer the JA signaling pathway to activate several defence mechanisms and hormone biosynthesis in plant cells [5,24,25]. Our RNA-seq data showed that 644 unigenes, including 226 DEGs, were annotated as having roles in the biosynthesis of plant hormones (Additional file 10).

Additionally, the annotation of RNA-seq data showed that 591 unigenes, including 204 DEGs, were involved in the biosynthesis of phenylpropanoids (Additional file 10). These compounds are used in plant defence to create physical and chemical barriers against infection, and as molecules involved in the local and systemic signaling of defence gene induction [27]. Unigenes related to key enzymes in the phenylpropanoid metabolism pathway [28] were differential expressioned in MeJA-treated *T. chinensis* cells and the mock-treated cells, such as L-phenylalanine ammonialyase (PAL), cinnamate 4-hydoxylase (C4H), 4-coumarate CoA ligase (4CL), chalcone synthase (CHS), chalcone flavavone isomerase (CFI), flavanone 3-hydroxylase (F3H) and dihydroflavonol reductase (DFR). These results suggest that MeJA affection of defence responses, hormone biosynthesis and phenylpropanoid biosynthesis identified in other plant species is also found in *T. chinensis* cells.

### MeJA-responsive transcription factors in *T. Chinensis* cells

Transcription factors (TFs) regulate the spatio-temporal expression of responsive genes to abiotic and biotic stresses in the defence mechanisms of plants [29,30]. Our sequence data showed that 450 unigenes were annotated to encode putative TFs (Additional file 12), including 57 up-regulated and 156 down-regulated genes (Additional file 13, Additional file 14). These 213 unigenes differentially expressed in response to MeJA elicitation were largely represented by the TF families regulating secondary metabolism and stress responses in plants, e.g. MYB superfamily (41 members), AP2 superfamily (40 members) and bHLH superfamily (27 members). The 10 most differentially up-regulated unigenes encoding TFs were SPL14 (*Arabidopsis thaliana*), HB-1(*A. thaliana*), MYC-like protein (*A. thaliana*), MYBJ6 (*Glycine max*), ERF2 transcription factor (*Brassica napus*), WOX8 (*A. thaliana*), AP2-EREBP (*Lotus japonicus*), heat stress transcription factor 12 (*Oryza sativa*) and two AP2/ERF (*Populus trichocarpa*). Abundant unigenes encoding putative TFs responding to MeJA elicitation showed that transcription regulation played a key role in MeJA-mediated taxol biosynthesis and the formation of MeJA-mediated response network in *T. chinensis* cells.

### MeJA increases terpenoid biosynthesis

Terpenoids are the most abundant and structurally diverse group of plant secondary metabolites and are derived from the universal precursor isopentenyl diphosphate (IPP) and dimethylallyl diphosphate (DMAPP) [31,32]. All known genes associated with IPP biosynthesis were mapped in the RNA-seq atlas of *T. chinensis* cells, and several unigenes respectively corresponding to these genes were present (Additional file 3). Some unigenes corresponding to geranyl diphosphate (GPP) synthase, farnesyl diphosphate (FPP) synthase and geranylgeranyl diphosphate (GGPP) synthase, which regulate the flux of terpenoid biosynthesis, were also present in the transcriptome data (Additional file 3). Additionally, our sequence data revealed that 737 unigenes, including 258 DEGs, were annotated as terpenoid biosynthesis pathway members. Among them, 371 unigenes, including 130 DEGs, were involved in the biosynthesis of terpenoids and steroids; 236 unigenes, including 63 DEGs, were involved in the biosynthesis of alkaloids derived from terpenoid and polyketide; 69 unigenes, including 33 DEGs, were involved in terpenoid backbone biosynthesis; 54 unigenes, including 29 DEGs, were involved in diterpenoid biosynthesis; and 7 unigenes, including 3 DEGs, were involved in monoterpenoid biosynthesis (Additional file 10). In summary, (1) an abundance of genes involved in terpenoid biosynthesis was present in the transcriptome of *T. chinensis* cells, (2) many genes involved in others secondary metabolic pathways, such as alkaloids and polyketides biosynthesis pathway were also found to be present in *T. chinensis*, and (3) the expression of genes involved in secondary metabolites in *T. chinensis* cells was induced by exogenous MeJA.

### Transcriptional control of taxane biosynthesis is regulated by MeJA elicitation

**Figure 6:**
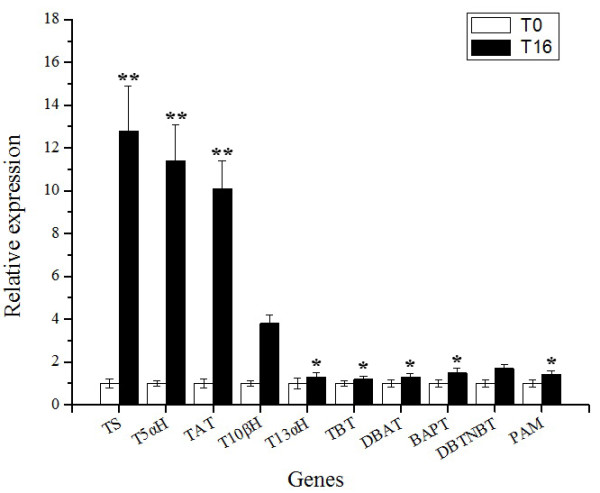
**Relative expression of 10 selected taxol biosynthesis genes as determined by qRT-PCR.** qRT-PCR analysis of 10 selected taxol biosynthesis genes, *P < 0.05, **P < 0.01. TS is short for taxadiene synthase, T5αH for taxane 5-alpha hydroxylase, TAT for 5-O-aceyltranferase, T10βH for taxane 10-beta hydroxylase, T13αH for taxane 13-alpha hydroxylase, TBT for 2-benzoyltransferase, DBAT for 10-O-aceyltransferase, BAPT for phenylpropanoyltransferase, DBTNBT for 3'-N-debenzoyltaxol N-benzoyltransferase, PAM for phenylalanine aminomutase.

All taxanes arise from geranylgeranylpyrophosphate (GGPP), a universal precursor for diterpene biosynthesis. About 19 biosynthetic enzymes are needed to form taxol from GGPP including taxadiene synthase (TS), eight cytochrome P450 oxygenases, three CoA-dependent acylations and several other enzymes [33-35], as well as several undefined enzymes in the taxol biosynthetic pathway [34-36]. Our sequence data showed that all of the previously defined genes for taxol biosynthesis were present in the transcriptome data of both T0 and T16 and the mRNA levels of most of them were higher in T16 than T0: unigenes corresponding to TS, T5αH, TAT and T7βH, T10βH were strongly up-regulated, unigenes corresponding to DBAT, PAM, DBTNBT and BAPT were slightly up-regulated, whereas unigenes corresponding to T2αH, T13αH and TBT were not distinctly up-regulated at 16 h after MeJA elicitation (Table 2). Gene expression of 10 selected taxol biosynthesis genes were validated by qRT-PCR (Figure 6). The results show that the expression profiles of these genes were consistent with the RNA-Seq data. These results clearly confirm that taxane biosynthesis is regulated by MeJA elicitation in *T. chinensis* cells, and further supported that the RNA-seq data is reliable.

**Table 2:** The putative genes involved in taxol biosynthesis

**Unigene ID**	**Annotation**	**RPKM_T0**	**RPKM_T16**
Unigene10976_All	taxadiene synthase (TS)	1.1866	6.592
Unigene40124_All	10-O-aceyltransferase (DBAT)	2.5128	6.2081
Unigene7834_All	5-O-aceyltranferase (TAT)	216.7005	1130.784
Unigene40964_All	2-benzoyltransferase (TBT)	1.1164	1.931
Unigene11678_All	taxane 10-beta hydroxylase (T10βH)	1.2889	22.6354
Unigene2856_All	taxane 13-alpha hydroxylase (T13αH)	7.4494	8.4546
Unigene39159_All	phenylpropanoyltransferase (BAPT)	2.1889	4.5731
Unigene18076_All	3'-N-debenzoyltaxol N-benzoyltransferase (DBTNBT)	2.6067	6.7517
Unigene6346_All	taxane 2-alpha hydroxylase (T2αH)	17.2825	27.2296
Unigene13946_All	taxane 5-alpha hydroxylase (T5αH)	2.3604	16.2127
Unigene35728_All	phenylalanine aminomutase (PAM)	0.3506	1.1578
Unigene46528_All	taxane 7-beta hydroxylase (T7βH)	0.4963	6.7533

Interestingly, we found that several unigenes were identified to correspond to hydroxylase, epoxidase, dehydrogenase and CoA ligase (Additional file 3), which provided candidates of presumptive biosynthetic enzymes for the remaining steps in taxol biosynthesis pathways such as C1β-hydroxylase, C9α-hydroxylase, cytochrome P450 C2’-side-chain hydroxylase, C4, C20-epoxidase, pyridine nucleotide-dependent dehydrogenase, and β-phenylalanoyl CoA ligase [33].

## Discussion

In *Taxus* cells, MeJA can effectively increase the production levels of taxol and other taxanes [2-4], although our understanding of the MeJA-mediated regulation of taxol biosynthesis is limited. Therefore, clarification of MeJA-mediated regulation mechanism of taxane biosynthesis and identification of genes whose regulation by MeJA is not yet recognised and genes whose products control taxane synthesis are needed.

Our results showed that *T. chinensis* cells 16 h after MeJA elicitation were characterized by maximum mRNA levels and all known genes involved in taxol biosynthesis were found, which was consistent with results reported in *T. cuspidata* cells under similar MeJA treatment conditions [33]. To investigate the molecular mechanisms of MeJA-mediated taxane biosynthesis and gain more information on MeJA-responsive genes, we used the MeJA-treated *T. chinensis* cells (T16) and the mock-treated cells (T0) for RNA-seq analysis to profile their transcriptome and transcriptional alterations in response to MeJA elicitation. More than 58 million sequence reads were generated and each of the two samples was represented by at least 29 million reads in which the tag density was sufficient for qualitative analysis of gene expression [22]. Due to the lack of available genome sequence for *Taxus sp.*, the sequence reads were not aligned to the reference genome to determine the genomic locations and distribution. We identified and annotated these sequences by using a series of bioinformatics tools to produce 46,581 unigenes including 13,469 differentially expressed in response to MeJA. All of the known genes involved in the JA biosynthetic and the JA signaling pathways could be identified, which suggested that the JA signaling pathway existed in the *T.chinensis* cells. Many studies had shown that application of exogenous MeJA induces the JA signaling pathway in several plants [26,27,37-40], including *Arabidopsis*, tobacco and *Catharanthus roseus*. In *Arabidopsis* cells, MeJA not only induces expression of genes encoding for JA biosynthetic enzymes [5], but induces the JA signaling pathway. A consistent aspect of induction of this pathway is the binding of the F-box protein CORONATINE INSENSITIVE 1 (COI1) to members of the JA ZIM domain (JAZ) protein family, which marks the complex for degradation by the 26S proteasome in the presence of JA-isoleucine (JA-Ile) and frees the basic helix–loop–helix (bHLH) transcription factor (TF) MYC2, which in turn helps regulate expression of a series of JA-inducible genes [25,39]. In this study with *T. chinensis* cells, our RNA-seq and qRT-PCR results showed that the expression of presumptive JA signaling pathway genes (COI1, JAZ, MYC2) was consistent with the mechanisms seen in other plants, suggesting exogenous application of MeJA could mediate JA biosynthesis and the JA signaling pathway, thereby regulating a series of downstream genes in *T. chinensis* cells.

In the present study, the known genes found in pathways that regulate taxol synthesis, as well as a large number of genes with known or predicted functions involved in several metabolic pathways, plant hormone biosynthesis and phenylpropanoid biosynthesis, as well as many genes encoding transcription factors were all shown to be induced in response to MeJA.

In plant cells, one major regulatory mechanism of secondary metabolite production is via the the control of the expression of transcription factors that in turn regulate biosynthesis genes [29,41], e.g. the ORCA3 transcription factor regulates several JA-responsive genes in MeJA-inducible indole alkaloid biosynthesis in *Catharanthus roseus*[42]. Similarly, stress responsive transcription factors have been suggested to be involved in taxol biosynthesis. Our sequence results also showed that many genes encoding transcription factors were differentially expressed in response to MeJA elicitation. These MeJA-responsive transcription factors may directly or indirectly regulate the production or activity of taxol biosynthetic enzymes; thus characterization of the DEGs which encode transcription factors might shed light on the regulation of taxol biosynthesis in *Taxus*.

Although structural elucidation of taxol has been extensively studied, taxol biosynthesis still needs to be further elucidated [34-36]. To better understand taxol biosynthesis and the regulation and origins of this pathway, Croteau et al. [33] identified genes encoding two previously uncharacterized cytochrome P450 taxoid hydroxylases and provided candidate genes for all but one of the remaining six steps by random sequencing of a *T. cuspidata* cDNA library from *T. cuspidata* cells 16 h after treatment with MeJA. By utilizing “pyrosequencing technology” with samples derived from the needles of *T. cuspidata*, Wu et al. [43] annotated about 14,095 unique sequences and identified candidates for the taxol biosynthetic genes from the needles of *T. cuspidata*. In addition, through a high throughput sequencing technology, Qiu et al. [44] found that *T. chinensis* cells have a complex and diverse small RNA population and exogenous MeJA significantly affected the expression of specific miRNAs in *T. chinensis* cells. Hao et al. [45] obtain a large number of unigenes by using illumina second generation sequencing in the study of tissue specific *Taxus* transcriptome. Our RNA-seq data also provided available candidate genes of presumptive biosynthetic enzymes for the all remaining steps in taxol biosynthesis pathway. The molecular cloning and characterization of these candidate genes will further elucidate the taxol biosynthesis pathway in *Taxus*.

## Conclusions

Using Illumina sequencing technology, we investigated the poly (A) + transcriptome of the MeJA-treated *T. chinensis* cells versus mock-treated cells and produced 46,581 assembled unigenes with 25,812 unigenes that could be annotated compared to other known genes from other plant species. Analysis of the annotated unigenes showed a significant transcriptional complexity in *T. chinensis* cells and provided more information about MeJA response. Genes encoding key enzymes in *T. chinensis* were identified and metabolic pathways involved in biosynthesis of JA, phenylpropanoids and terpenoid were bioinformatically reconstructed in *T. chinensis*. Additionally, the nucleotide sequences obtained through transcriptome sequencing serves as a substantial contribution to existing sequence resources of *T. chinensis*. Particularly, the transcriptome data provided candidates of presumptive biosynthetic enzymes for the remaining steps in taxol biosynthesis pathways. Further analysis of the *T. chinensis* genes annotated to transciption factors will help us understand regulation patterns upon MeJA elicitation and the molecular mechanisms of MeJA-mediated taxane biosynthesis in *Taxus* cells. In summary, this transcriptome data will serve as an important public information platform to accelerate research of MeJA-responsive networks and the regulatory mechanisms of taxol biosynthesis.

## Methods

### Plant samples, RNA isolation, cDNA synthesis, and sequencing

Cultured suspension cells of *Taxus chinensis* (Pilger) Rehd cell line 48# were established from callus cultures initiated embryos excised from leaves collected in Wuhan (Hubei province, China) in May 2003, and maintained with modified Gamborg’s B5 medium as previously described [3]. For methyl jasmonate (MeJA) elicitation, a 7-day-old culture was diluted 10-fold in fresh medium and grown for 7 d in the dark at 26 °C. Subsequently, MeJA (Sigma, USA) at a final concentration of 100 μM or an equal volume of solvent as a control was added to the cultures. For the transcriptome analysis, samples were taken at 16 h after MeJA addition. The treated cells were collected and immediately stored in liquid nitrogen until total RNA was isolated. Total RNAs of *Taxus* cells were isolated with TRIzol reagent (Invitrogen, USA) according to manufacturer’s protocol. The RNA samples were treated with DNase I (Invitrogen) and then sent to the Beijing Genomics Institute-Shenzhen (BGI, Shenzhen, China) for mRNA purification, cDNA library construction and sequencing using the Illumina/Solexa technology.

### Sequence analysis

Transcriptome *de novo* assembly was carried out with short reads assembling program SOAPdenovo which applies de Bruijn graph algorithm [18]. After assessing different K-mer sizes, we found that K-mer size of 21 achieved the best balance between the number of contigs produced, coverage and average sequence length attained. SOAPdenovo was used to combine reads with certain length of overlap to form longer fragments (contigs) and then the reads were mapped back to contigs; with paired-end reads it is able to detect contigs from the same transcript as well as the distances between these contigs. Next, SOAPdenovo was used to connect the contigs using N to represent unknown sequences between each two contigs to produce scaffolds.

Paired-end reads were used again for gap filling of scaffolds to get sequences with the least Ns and that could not be extended on either end. Such sequences were defined as unigene sequences. The transcript sequences from each of the two sample’s assembly were further spliced and redundancy was removed with the sequence clustering software-TGICL [19] to acquire non-redundant transcript sequences of maximum length. Finally, blastx alignment (evalue < 0.00001) between transcript sequences and protein databases such as NR, Swiss-Prot, KEGG and COG was performed, and the best aligning results were used to decide sequence direction of unigene sequences.

### Identification of differentially expressed genes

Firstly, normalized expression value of genes were calculated by a RPKM (Reads Per kb per Million reads) method [22], which was denoted as a formula:$RPKM=\frac{10^{6}C}{NL/10^{3}}$. RPKM represented the expression level of a given unigenes sequence, C represents the number of reads uniquely aligned to this given transcript sequence, N represents total number of reads that uniquely aligned to all transcript sequences, and L represents the number of bases on this given transcript sequence. We used the absolute value of log2Ratio represents the multiples of differential expression between two samples.

Then, we identified differentially expressed genes between two samples by FDR (False Discovery Rate) method according to Audic et al. [23].

The probability of this given transcript sequence expressed equally between T0 and T16 samples can be calculated with the following formula:

(1) $$2\sum_{i=0}^{i=y}p\left(i|x\right)$$

or $2\times \left(1-\sum_{i=0}^{i=y}p\left(i|x\right)\right)\left(\mathrm{if}\cdot^{\sum_{i=0}^{i=y}p\left(i|x\right)>0.5}\right)\text{,}$Wherein $p\left(y|x\right)=\left(\frac{N_{2}}{N_{1}}\right)y\frac{(x+y)!}{x!y!{\left(1+\frac{N_{2}}{N_{1}}\right)}^{\left(x+y+1\right)}}$N1 represents the total clean tag number of T0, N2 represents the total clean tag number of T16; x represents the tag number of gene A in T0 and y represents the tag number of gene A in T16. P value corresponds to the differential gene expression test.

FDR is a method to determine the appropriate threshold of the P value in multiple test and analysis. Assuming that we have picked out R presumptively differentially expressed genes in which S genes truly show differential expression and the other V genes are false positives. If we decide that the error ratio “Q = V/R” must stay below a cutoff (e.g. 5%), we should preset the FDR to a number no larger than 0.05.

Finally, we used FDR ≤ 0.001 and the absolute value of log2Ratio ≥ 1 as the threshold to judge the significance of gene expression difference.

### Real-time PCR validation

Quantitative Real-Time PCR (qRT-PCR) was run using SYBR premix Ex Taq Kit (Takara, Japan) and ABI PRISM 7700 DNA Sequence Detection System (Applied Biosystems, USA) using the same cDNA samples as used with the RNA-seq experiment. A first-strand cDNA fragment was synthesized from total RNAs treated with DNAase-I (Invitrogen) using Superscript II reverse transcriptase (Invitrogen). Gene-specific primers were designed for target transcript sequences and 18*S* rDNA sequence as an internal control (Additional file 15). The comparative threshold cycle method was used to calculate the relative gene expression [46]. Each real-time PCR was carried out three times.

## Competing interests

The authors declare that they have no competing interests.

## Authors’ contributions

Conceived and designed the experiments: L-jY, PZ, C-hF, C-fZ and S-tL. Performed the experiments: S-tL, PZ, MZ. Analyzed the data: C-hF, S-tL, MZ, PZ, C-fZ, A-yG. Contributed reagents/materials/analysis tools: L-jY, C-hF, C-fZ, Y-sD. Wrote the paper: S-tL, PZ, MZ, C-hF, C-fZ and L-jY. All authors read and approved the final manuscript.

## Supplementary Material

Additional file 1 Contigs length distribution of T0 and T16. Click here for file

Additional file 2 Scaffolds length distribution of T0 and T16. Click here for file

Additional file 3 The annotation of all unigenes. Click here for file

Additional file 4 Unigenes with pathway annotation. Click here for file

Additional file 5 Overview of up-regulated genes. Click here for file

Additional file 6 Overview of down-regulated genes. Click here for file

Additional file 7 GO annotation of DGEs. Click here for file

Additional file 8 GO categories for up- and down-regulated genes. Click here for file

Additional file 9 The KEGG analyses separated by cell line. Click here for file

Additional file 10 DEGs with pathway annotation. Click here for file

Additional file 11 Relative expression of randomly selected genes as determined by qRT-PCR. Click here for file

Additional file 12 All transcript sequences identified to encode putative factors. Click here for file

Additional file 13 The up-regulated transcript sequences encoding putative transcription factors. Click here for file

Additional file 14 The down-regulated transcript sequences encoding putative transcription factors. Click here for file

Additional file 15 Primers used in qRT-PCR for validation of differentially expressed genes. Click here for file
