# Supplementary figures and images for: Transcriptional profile of Taxus chinensis cells in response to methyl jasmonate
Source: BMC Genomics. 2012 Jul 2;13:295. doi: 10.1186/1471-2164-13-295 (PMC3414795; doi:10.1186/1471-2164-13-295)

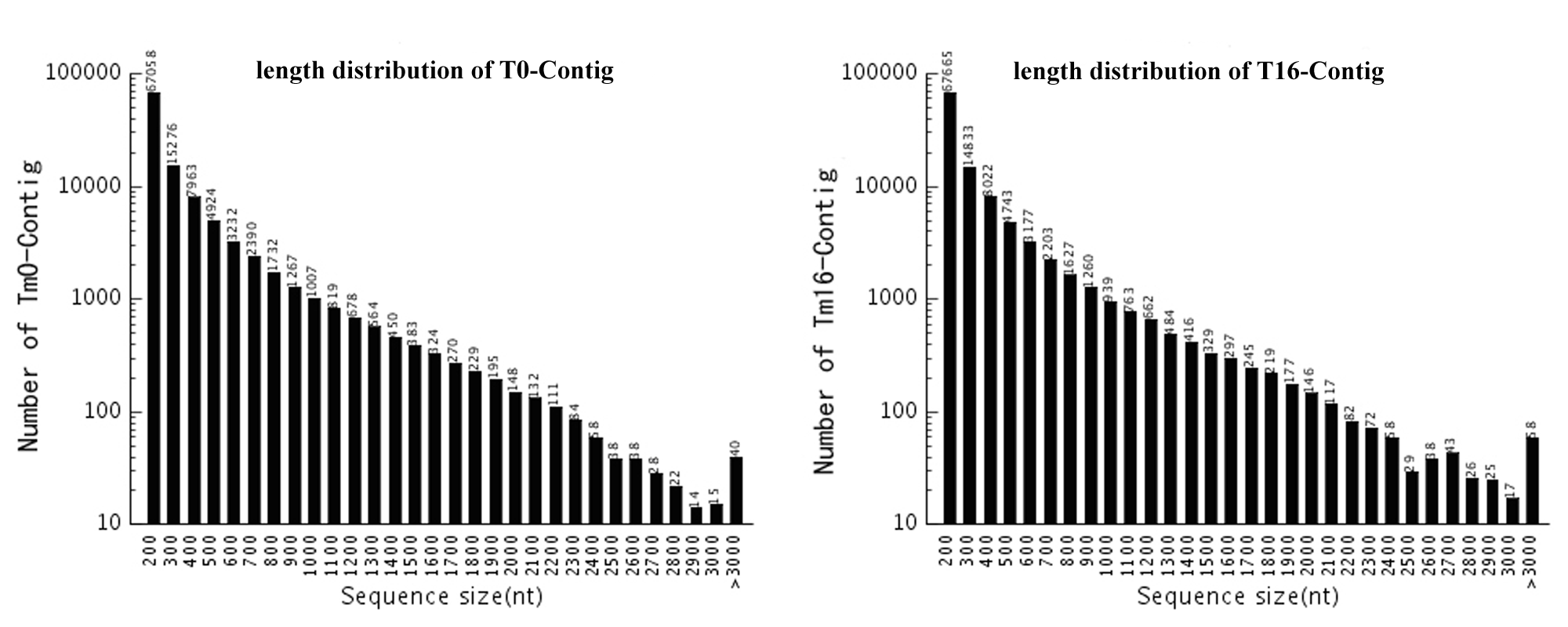

Supplement: Additional file 1 — Contigs length distribution of T0 and T16. [file 1471-2164-13-295-S1.tiff]

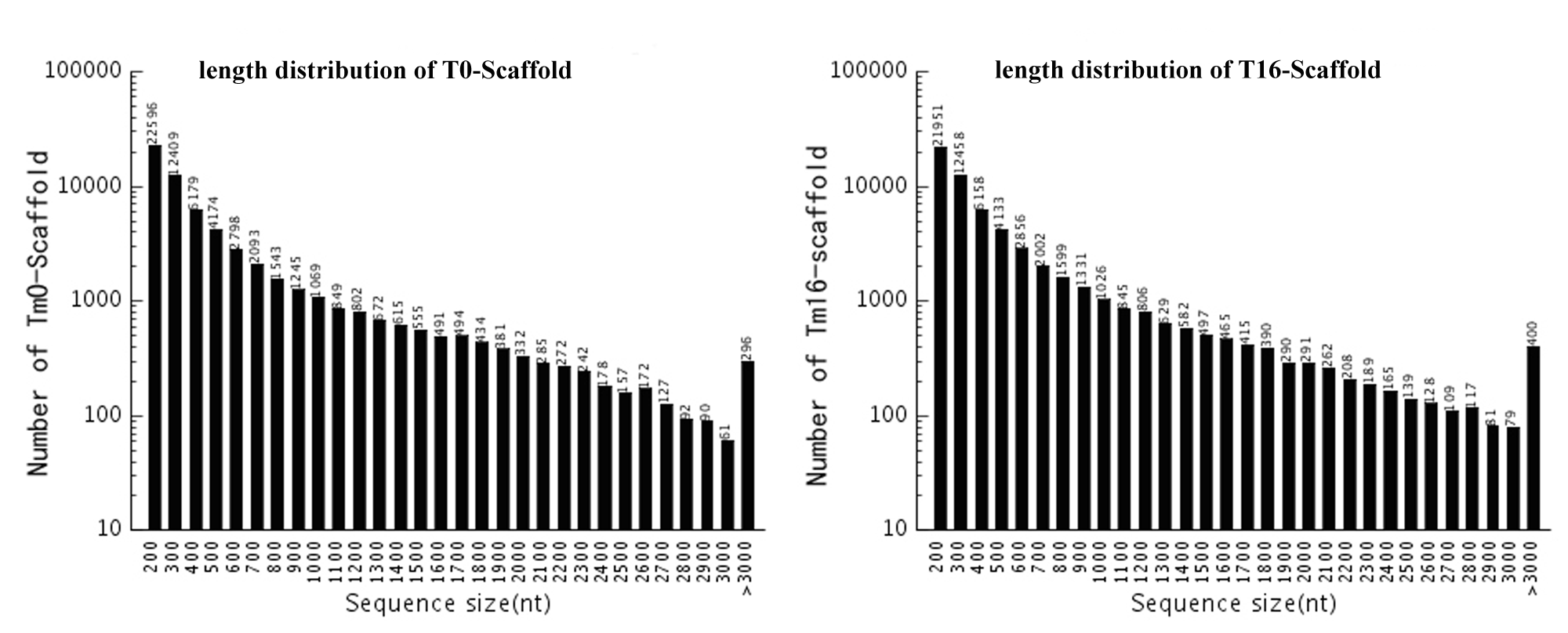

Supplement: Additional file 2 — Scaffolds length distribution of T0 and T16. [file 1471-2164-13-295-S2.tiff]

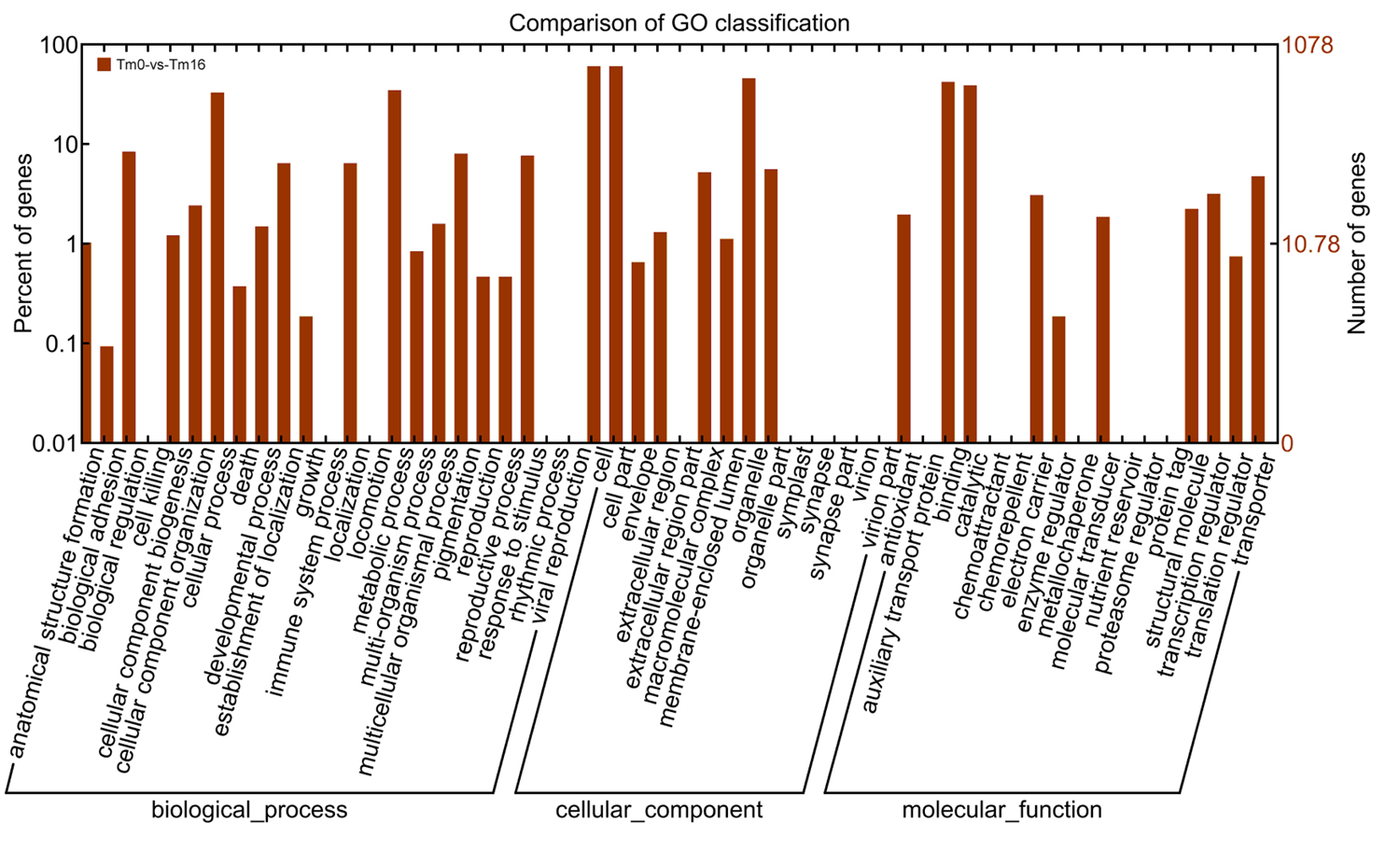

Supplement: Additional file 7 — GO annotation of DGEs. [file 1471-2164-13-295-S7.tiff]

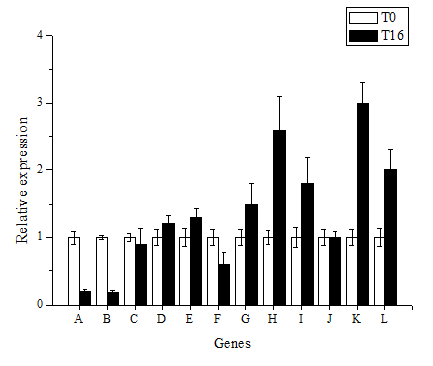

Supplement: Additional file 11 — Relative expression of randomly selected genes as determined by qRT-PCR. [file 1471-2164-13-295-S11.tiff]
